# Mechanistic Role of Sestrin2 in Exercise-Mediated Cardioprotection Against Obesity-Related Cardiomyopathy

**DOI:** 10.3390/ijms27135670

**Published:** 2026-06-23

**Authors:** Meili Hao, Wanyu Zhu, Li Zhao, Wenyan Bo

**Affiliations:** 1 School of Physical Education, Luoyang Normal University, Luoyang 471934, China; haomeili@lynu.edu.cn (M.H.); zhaoli@lynu.edu.cn (L.Z.); 2 School of Physical Education, Shaanxi Normal University, Xi’an 710119, China; zhuwanyu2000@126.com; 3 School of Physical Education, Shanxi University, Taiyuan 030006, China

**Keywords:** Sestrin2, aerobic exercise, cardiac function, inflammation, oxidative stress

## Abstract

Exercise is one of the safe and effective methods to improve obesity and its complications, but the mechanism has not been fully elucidated. Sestrin2 (SESN2) is a stress-induced protein that protects cells from stress damage. The role and mechanism of SESN2 in the improvement of obesity-induced cardiac dysfunction by exercise are still unclear. Male C57BL/6J mice were used to prepare a high-fat diet-induced obesity mouse model and conducted aerobic exercise training. After training, echocardiography was used to evaluate the cardiac function of mice, and HE and Masson staining were used to assess the extent of cardiac damage. Cell experiments were conducted using the H9C2 cell line derived from embryonic rat hearts, with the intervention of palmitic acid ester and exogenous SESN2. We detected indicators related to myocardial cell damage, fibrosis, inflammation, and oxidative stress, as well as the activation level of the AMPK-PGC-1α signaling pathway. The results showed that aerobic exercise significantly inhibited myocardial fibrosis, inflammation, oxidative stress, and cell damage in HFD mice, upregulated cardiac SESN2 expression, and activated the AMPK-PGC-1α signaling pathway. Cell experiments have found that exogenous SESN2 pretreatment alleviates palmitate-induced injury, inflammation, and oxidative stress in H9C2 cardiomyocytes, and activates the AMPK-PGC-1α signaling pathway. This indicates that aerobic exercise significantly upregulates the expression of SESN2 and activates the AMPK-PGC-1α signaling pathway, which is potentially involved in alleviating myocardial inflammation, oxidative stress, cardiac fibrosis and cardiac dysfunction in HFD mice.

## 1. Introduction

In the context of the rapid advancement of the global economy and the transformation of lifestyles, obesity has manifested as a significant public health challenge, mainly propelled by excessive caloric consumption and physical inactivity [1]. Obesity is closely associated with a range of chronic diseases, including hypertension, diabetes mellitus, and cardiovascular diseases, significantly reducing life expectancy [2,3]. Notably, obesity can directly induce cardiac remodeling and dysfunction even in the absence of comorbidities such as coronary artery disease or hypertension, a condition referred to as obesity-related cardiomyopathy. This pathological process is characterized by myocardial fibrosis and impaired ventricular contractility, and may ultimately progress to heart failure or sudden cardiac death [4,5,6,7]. Accumulating evidence indicates that long-term exercise not only mitigates obesity and improves insulin resistance [8], but also exerts beneficial effects on obesity-induced cardiac dysfunction [9]. Exercise training has been shown to attenuate systemic low-grade inflammation in overweight and obese individuals [10], and animal studies further demonstrate its capacity to reduce oxidative stress and inflammatory responses in the myocardium of diet-induced obese mice [11]. Despite these well-documented benefits, the precise molecular mechanisms underlying the cardioprotective effects of exercise in the context of obesity remain incompletely understood.

Sestrin2 (SESN2), a stress-inducible protein, plays a critical role in cellular defense against metabolic and oxidative stress. Reduced SESN2 expression has been linked to oxidative damage, mitochondrial dysfunction, impaired glucose tolerance, and the development of diabetes [12]. Clinical studies have reported significantly decreased SESN2 levels in obese individuals [13,14]. However, there are currently no published literature reports on the changes in SESN2 in obese adults. In experimental models, *sesn2* overexpression has been shown to alleviate obesity-related cardiac injury by reducing reactive oxygen species (ROS) accumulation and myocardial fibrosis, thereby improving cardiac function [15]. SESN2 attenuates cardiac endoplasmic reticulum stress post-Ischemia/Reperfusion injury via regulation of mTOR signaling [16]. SESN2 protected against doxorubicin-induced cardiotoxicity through improving mitochondria function and mitophagy [17]. Furthermore, SESN2 confers cardioprotection against ischemic injury through activation of the AMPK–PGC-1α signaling pathway [18]. Emerging evidence suggests that exercise can upregulate SESN2 expression in an AMPKα2-dependent manner and improve insulin sensitivity [19]. Aerobic exercise inhibited myocarditis cytokines by upregulating SESN2through the AMPK-PGC-1α pathway, increased peptide hormone mRNA levels, and played a role in defending against lipotoxic myocardial injury [20]. However, the role of SESN2 in mediating exercise-induced improvements in obesity-related cardiac dysfunction has not yet been elucidated. Therefore, the present study aims to investigate the functional role and underlying mechanisms of SESN2 in exercise-mediated protection against obesity-induced cardiac dysfunction, thereby providing novel insights into potential therapeutic strategies for obesity-related cardiomyopathy.

## 2. Results

### 2.1. Exercise Significantly Ameliorated Myocardial Injury in HFD-Induced Obese Mice

**Figure 1:**
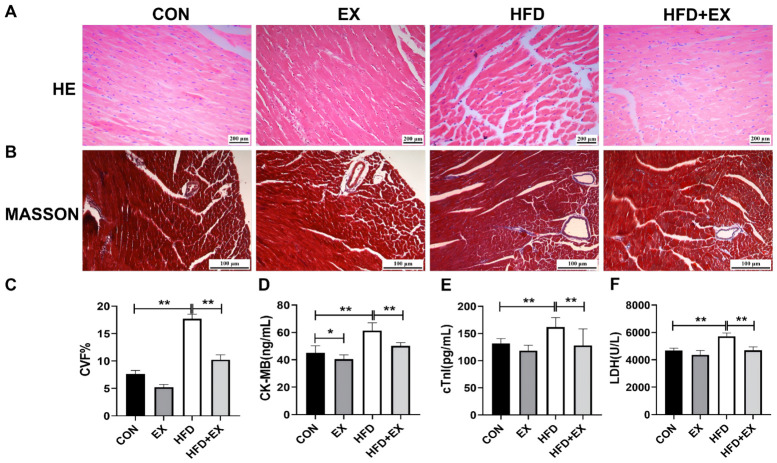
Diet-induced obesity leads to cardiac damage. (**A**). Hematoxylin and eosin (HE) staining images of myocardial cells (20× magnification). (**B**). For Masson staining, red represents the cytoplasm, blue represents collagen fibers, and brown represents nuclei. (**C**). Myocardial collagen volume fraction. CVF% = (collagen area/total myocardial area) × 100%. (**D**). CK-MB content in serum. (**E**). cTnI content in serum. (**F**). LDH content in serum. Values are mean ± SEM. * *p* < 0.05, ** *p* < 0.01, *n* = 6.

HE staining of heart tissue revealed distinct morphological differences among groups. Masson staining was used to detect the collagen accumulation in heart tissue, and described using the myocardial collagen volume fraction. Compared with the CON group, HFD mice showed disordered and fragmented myocardial fibers, a significantly increased cross-sectional area of myocardial cells, and significantly increased myocardial collagen fibers (Figure 1A–C). Serum CK-MB, LDH, and cTnI levels, cardiac injury markers, were elevated (Figure 1D–F), indicating that obesity induced cardiac injury, which was significantly reversed after exercise intervention (Figure 1D–F). Echocardiography results showed that EF and FS were decreased in HFD mice, and the exercise intervention significantly increased EF and FS in HFD mice. These results suggested that left ventricular systolic function is significantly impaired in high-fat diet-induced obese mice, and the aerobic exercise intervention significantly improved cardiac function (Table 1).

### 2.2. Exercise Prevented Cardiac Inflammation in HFD-Induced Obese Mice

**Figure 2:**
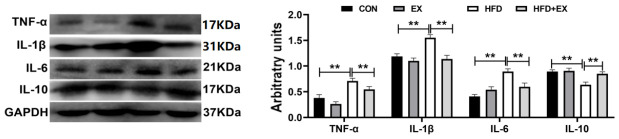
Exercise reduced HFD-induced heart inflammation. Representative Western blot images of TNF-α, IL-6, IL-1β, and IL-10 in myocardium from the CON, EX, HFD and HFD + EX groups. GAPDH served as the loading control. Quantification of protein expression levels was normalized to GAPDH. Values are mean ± SEM. ** *p* < 0.01, *n* = 6.

It is well known that HFD induces many inflammatory responses in the heart, mediating the pathogenesis of cardiac injury and remodeling. Therefore, we assessed the level of inflammation in the heart. The results showed that compared with the CON group, high-fat fed mice had their heart TNF-α, IL-1β, and IL-6 expression significantly increased and IL-10 expression significantly decreased, and exercise intervention significantly reversed these changes (Figure 2). This result suggested that aerobic exercise significantly reduced high-fat diet-induced cardiac inflammation in obese mice.

### 2.3. Exercise Improved Cardiac Oxidative Stress Induced by HFD in Obese Mice

**Figure 3:**
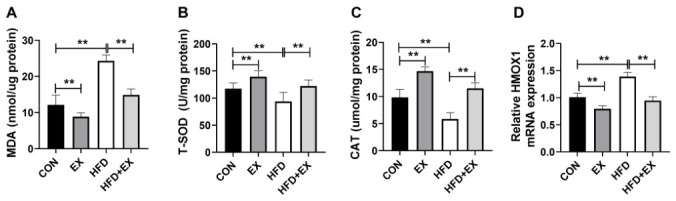
Exercise alleviated HFD-induced cardiac oxidative stress. (**A**). The MDA content. (**B**). T-SOD activity. (**C**). CAT activity. (**D**). Cardiac *hmox1* mRNA levels determined with qPCR. Values are mean ± SEM. ** *p* < 0.01, *n* = 6.

Studies have shown that hyperlipidemia can hyperpolarize mitochondria, leading to electron slip and enhanced ROS production, leading to cardiac damage and obesity-related complications [21]. The free radical scavenging enzyme SOD eliminates excess ROS during cardiac injury. MDA is a biomarker of oxidative stress. Compared with the CON group, the content of MDA was significantly decreased, the activities of T-SOD and CAT were increased, and *hmox1* mRNA levels were significantly decreased in the EX group. In the HFD group, the MDA content was significantly increased, T-SOD and CAT activities were significantly decreased, and *hmox1* mRNA levels were significantly increased when compared with the CON group. Compared with the HFD group, the MDA content was significantly decreased, T-SOD and CAT activities were significantly increased (Figure 3A–C), and *hmox1* mRNA levels were significantly decreased in the HFD + EX group (Figure 3D). In conclusion, aerobic exercise significantly reduced cardiac oxidative stress in HFD mice.

### 2.4. Exercise Significantly Improved SESN2 Expression and Activated the AMPK-PGC-1α Pathway in HFD Mice

**Figure 4:**
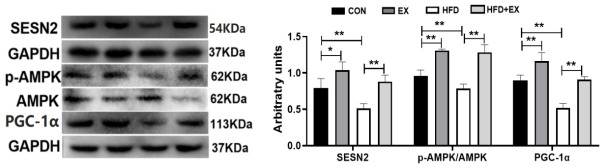
Exercise activated the SESN2-AMPK-PGC-1α pathway in HFD mice. Representative Western blot images of SESN2, p-AMPK, AMPK, PGC-1α in the myocardium from the CON, EX, HFD and HFD + EX groups. GAPDH served as the loading control. Quantification of protein expression levels were normalized to GAPDH. Values are mean ± SEM. * *p* < 0.05, ** *p* < 0.01, *n* = 6.

Western blot analysis revealed pronounced alterations in SESN2 and the AMPK-PGC-1α pathway in the myocardium of HFD mice. Compared with the CON group, aerobic exercise significantly upregulated the expression of myocardial SESN2 and activated the AMPK/PGC-1α signaling pathway, while the HFD group significantly reduced the expression of cardiac SESN2 and inhibited the AMPK/PGC-1α signaling pathway in mice. Compared with the HFD group, the HFD + EX group showed upregulation of myocardial SESN2 expression and activation of the AMPK/PGC-1α signaling pathway (Figure 4). This suggested that exercise may exert a protective effect on the heart of HFD mice by upregulating the expression of SESN2 and activating the AMPK-PGC-1α pathway.

### 2.5. Pretreatment with Exogenous SESN2 Attenuated Palmitate-Induced H9C2 Cardiomyocyte Injury and Inflammation

**Figure 5:**
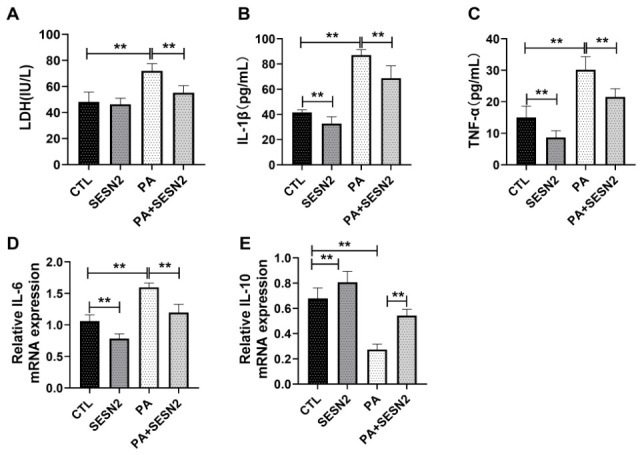
Exogenous SESN2 pretreatment alleviated PA-induced cardiomyocyte injury and inflammation. (**A**). The content of LDH. (**B**,**C**). The release of IL-1β and TNF-α was detected by kits. (**D**,**E**). Cardiac IL-6 and IL-10 mRNA levels determined with qPCR. Values are mean ± SEM. ** *p* < 0.01.

To further test whether the protective effect of exercise on HFD-induced cardiac injury was mediated via SESN2, we used palmitate (PA)-treated H9C2 cells to mimic fatty acid stimulation and pretreated them with exogenous SESN2. It was found that PA treatment significantly increased the release of LDH, IL-β, and TNF-α, as well as IL-6 and IL-10 mRNA expression in H9C2 cells, while exogenous SESN2 pretreatment significantly reversed these changes (Figure 5A–E). These results indicated that exogenous SESN2 significantly inhibited PA-induced H9C2 cell injury and inflammation.

### 2.6. Exogenous SESN2 Pretreatment Alleviated Palmitate-Induced Oxidative Stress in H9C2 Cardio-Myocytes

**Figure 6:**
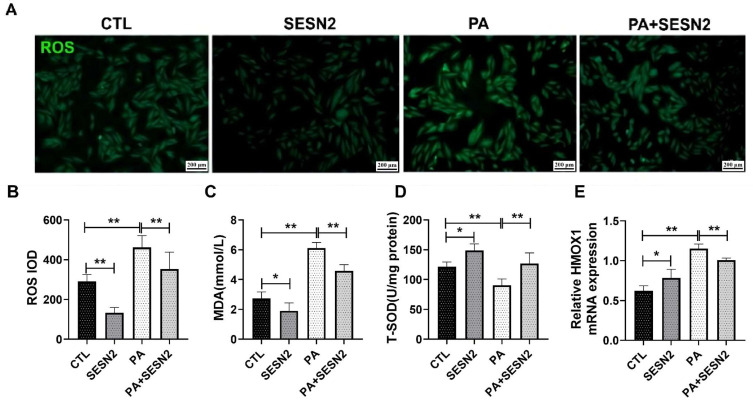
SESN2 alleviated PA-induced oxidative stress in H9C2 cells. (**A**). ROS immunofluorescence staining was performed. (**B**). Integrated Optical Density (IOD) of ROS. (**C**,**D**). The content of MDA and the activity of T-SOD were detected by kits. (**E**). *hmox1* mRNA levels determined with qPCR. Values are mean ± SEM. * *p* < 0.05, ** *p* < 0.01.

ROS staining showed that SESN2 pretreatment significantly inhibited the ROS elevation induced by PA stimulation in H9C2 cells (Figure 6A,B). Meanwhile, pretreatment with SESN2 significantly inhibited the increase of MDA content (Figure 6C), the decrease of T-SOD activity and the increase of *hmox1* mRNA levels (Figure 6D,E) in H9C2 cells induced by PA stimulation. These results indicated that SESN2 pretreatment significantly inhibited the oxidative stress induced by PA stimulation in H9C2 cells.

### 2.7. Exogenous SESN2 Activated the AMPK-PGC-1α Signaling Pathway in H9C2 Cells

**Figure 7:**
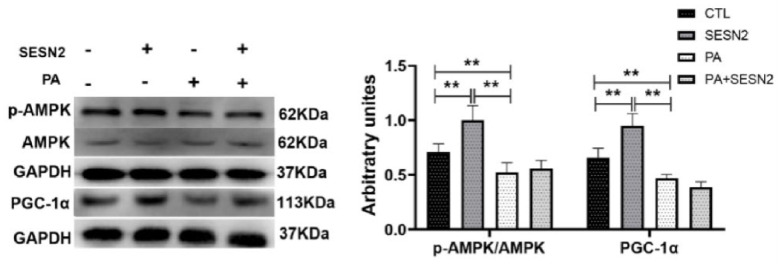
SESN2 alleviated the AMPK-PGC-1α signaling pathway in H9C2 cells. Representative Western blot images of SESN2, p-AMPK, AMPK, and PGC-1α in H9C2 cells. GAPDH served as the loading control. Quantification of protein expression levels were normalized to GAPDH. Values are mean ± SEM. ** *p* < 0.01.

To verify the mechanism of action of SESN2, we administered exogenous SESN2 intervention and PA stimulation to H9C2 cells. The results showed that PA intervention significantly inhibited the AMPK/PGC-1α signaling pathway, while SESN2 intervention significantly rescued this inhibitory effect (Figure 7). Therefore, it indicated that SESN2 rescued PA-induced H9C2 cell damage by inhibiting the AMPK/PGC-1α signaling pathway.

## 3. Discussion

Obesity has been recognized as a cause of various forms of cardiac abnormalities and the development of heart failure [22,23]. The main novel finding in our study was that exercise significantly ameliorated HFD-induced cardiac fibrosis, inflammation, and oxidative stress, which may be associated with the activation of the SESN2-AMPK-PGC-1α signaling pathway. In vitro studies showed that SESN2 pretreatment inhibited PA-induced inflammation, oxidative stress, and activating the AMPK-PGC-1α pathway in H9C2 cells. Our findings suggested that SESN2 may be one of the key targets responsible for the protective effect of aerobic exercise against diet-induced obesity-induced cardiac injury and shed light on future SESN2-based therapeutic interventions for obesity-related cardiac abnormalities.

Obesity is characterized by a chronic low-grade inflammatory state, which is widely recognized as a key pathological driver of cardiovascular dysfunction [24,25].This persistent inflammatory milieu contributes directly to the cardiac structural and functional abnormalities observed in obesity and related cardiovascular diseases [26,27]. Concurrently, enhanced oxidative stress further amplifies this process by promoting the production and release of pro-inflammatory cytokines, thereby accelerating the progression of obesity-associated complications [28,29]. Our experiments found that HFD mice had significantly increased expression of pro-inflammatory factors, decreased expression of anti-inflammatory factors, and increased cardiac oxidative stress levels. Sustained activation of inflammatory signaling pathways has been shown to induce cardiac fibrosis, a hallmark of adverse myocardial remodeling [30,31]. During cardiac stress, increased recruitment and activation of inflammatory cells, such as neutrophils and monocytes, initiate a cascade of inflammatory responses. Prolonged inflammation subsequently promotes fibroblast activation and extracellular matrix deposition, ultimately leading to myocardial fibrosis [32]. Consistent with previous reports demonstrating myocardial fibrosis in obese individuals [30,33], which is closely associated with ventricular diastolic dysfunction [34,35], we found that cardiac fibrosis was increased in HFD mice, with disordered cell distribution and increased secretion of myocardial injury markers. Moreover, echocardiographic assessment showed a significant reduction in EF, indicating impaired cardiac function. Collectively, these results demonstrate that HFD induces substantial cardiac injury and dysfunction, likely mediated by inflammation- and oxidative stress-driven fibrotic remodeling.

Exercise has been widely recognized as an effective non-pharmacological strategy to mitigate obesity-related comorbidities, improve cardiovascular health, and modulate inflammatory responses. Clinical studies have shown that appropriate exercise interventions reduce systemic inflammation in obese children and adolescents [36], while experimental evidence further indicates that exercise training attenuates oxidative stress and inflammatory responses in diet-induced obesity models [11,37]. In line with these findings, our results demonstrate that exercise significantly alleviated myocardial fibrosis, reduced inflammatory and oxidative stress levels, and improved cardiac function, as evidenced by the increased EF in HFD mice. These findings suggest that exercise effectively reverses HFD-induced cardiac remodeling and dysfunction, highlighting its therapeutic potential in the prevention and management of obesity-related cardiomyopathy.

It has been found that SESN2 expression is reduced in obese hearts, and knockout of SESN2 aggravates obesity-induced cardiac dysfunction [15]. SESN2 activated AKT/GSK-3β/NRF2 to ameliorate adriamycin-induced cardiotoxicity in obese mice induced by a high-fat diet [38]. These results suggest that SESN2 expression is associated with obesity-related myocardial injury. SESN2 has an antioxidant effect [14,31]. In addition, SESN2 exerted ischemic heart protection by activating the AMPK-PGC-1α pathway [18]. SESN2 overexpression prevents cell damage, inflammation and oxidative stress under HFD diet-induced obesity [15]. Acute resistance exercise increases SESN2 phosphorylation in adult skeletal muscle [39]. Animal experiments have shown that resistance exercise increased SESN2 protein content [40], and exercise increases SESN2 levels in an AMPKα2-dependent manner and improves insulin resistance [19]. SESN2 is a stress-induced protein and its expression increases due to stress, like ischemia. In our study, we found that cardiac SESN2 expression was significantly upregulated after aerobic exercise intervention, and the expression of SESN2 was negatively correlated with cardiac inflammation and oxidative stress. The discrepancy arises from the distinct stress types: HFD-induced chronic metabolic stress suppresses SESN2 expression via chronic inflammation and oxidative stress, while acute stress (like ischemia) induces SESN2 as a protective response. Moreover, we systematically clarify the molecular mechanism underlying the interaction between SESN2 and the AMPK/PGC-1α pathway. SESN2 exerts a regulatory effect on AMPK by modulating its kinase activity and phosphorylation level. Upon AMPK activation, downstream PGC-1α is significantly induced, which acts as a master transcriptional coactivator to mediate a series of downstream biological processes. Therefore, we hypothesized that exercise ameliorates diet-induced obese cardiac dysfunction by upregulating SESN2. We further validated this in cell experiments and found that exogenous SESN2 intervention significantly inhibited PA-induced cardiomyocyte injury, inflammation, and oxidative stress. Therefore, SESN2 may be one of the important targets for exercise to improve cardiac dysfunction in diet-induced obesity. All in all, our results found that aerobic exercise cardioprotection in obese mice may be associated with upregulation of SESN2 expression and activation of the AMPK-PGC-1α signaling pathway.

Several limitations of this study should be acknowledged. First, while we observed that exogenous SESN2 treatment protected H9C2 cells against palmitate-induced injury, we did not provide direct evidence that SESN2was taken up into the cells. As SESN2 is a non-secreted intracellular protein without known transmembrane domains, it is not inherently membrane-permeable. Whether the observed protective effects resulted from intracellular uptake (via endocytosis or macropinocytosis), cell surface binding to an unknown receptor, or indirect extracellular effects remains unknown. Future studies using fluorescently labeled SESN2 or endocytosis inhibitors are warranted to clarify the mechanism of SESN2–cell interaction. Second, all cellular experiments were performed using only H9C2 cardiomyocytes. A single cell model cannot fully recapitulate the complex microenvironment of tissues and organs in vivo. Future investigations using multiple cell lines, primary cells and animal models are required to further verify our conclusions. Third, the present study was conducted exclusively in male mice. Given that female mice exhibit relative protection from HFD-induced obesity during the early feeding period due to the effects of estrogen on energy homeostasis, it remains possible that the therapeutic efficacy of SESN2 may differ between sexes. Future studies using both male and female mice, including ovariectomized female mice to control for hormonal cycles, are necessary to determine whether SESN2 exerts similar metabolic protection in females. In addition, the detailed upstream and downstream regulatory molecules of the SESN2/AMPK/PGC-1α pathway remain to be explored in depth in subsequent work.

## 4. Materials and Methods

### 4.1. Experimental Animal Grouping and Exercise Protocol

Twenty-four 8-week-old C57BL/6J male mice were purchased from Xi’an Jiaotong University, Xi’an, China, and all experiments met NIH animal welfare standards. After one week of acclimatization, the mice were randomly allocated into four groups (*n* = 6 per group): the control group (CON), treadmill aerobic exercise group (EX), high-fat diet group (HFD), and high-fat diet and treadmill aerobic exercise group (HFD + EX). The CON and EX groups were fed a normal diet (1.8 kJ/g, 13% of energy as fat), and the HFD and HFD + EX groups were fed a high-quality diet (14.7 kJ/g, 60% of energy as fat). After the experiment, mice were anesthetized with 5% isoflurane and euthanized by bloodletting. This study is performed in accordance with the relevant guidelines and regulations. All methods are reported in accordance with the ARRIVE guidelines.

**Figure 8:**
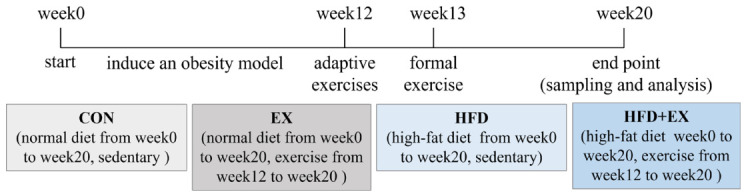
Experimental group diagram.

After 12 weeks of a high-fat diet, HFD mice exhibited a 20% increase in body weight relative to controls, confirming successful obesity model establishment. Then, mice in the EX and HFD + EX groups underwent treadmill aerobic exercise; during the first week, they engaged in adaptive exercises (8 m/min, 5 d/week), and then with formal exercise for 8 weeks (14 m/min, 60 min/d, 6 d/week). Mice performed one block of 60-min treadmill exercise per day. If mice exhibited signs of fatigue (such as failure to keep pace or dyspnea), they were permitted a 2–3-min rest and then resumed the exercise. Mice in the CON and HFD groups were not subjected to exercise training and were maintained under sedentary conditions. The experimental design is illustrated in Figure 8.

### 4.2. Echocardiography

During echocardiography, mice were placed on a temperature-controlled heated pad (37 °C, monitored via rectal probe). Anesthesia was maintained with 1.5% isoflurane, and heart rate was monitored throughout. Cardiac function was evaluated via transthoracic echocardiography using a Vetus 7 veterinary color Doppler ultrasound system (Mindray, Shenzhen, China). Parasternal short-axis M-mode images were measured and acquired by echocardiography, including left ventricular internal dimension diastolic (LVIDd), left ventricular internal dimension systole (LVIDs), fractional shortening (FS) and ejection fractions (EF), which were used to assess cardiac function.

### 4.3. HE and Masson Staining

Twenty-four hours after the end of training, the hearts were removed from the chest under 5% isoflurane anesthesia, washed with precooled PBS, and fixed in 4% neutral formaldehyde. After treatment in graded alcohol and xylene, hearts were embedded in paraffin and then serially sectioned (5 μm). To observe the myocardial morphology and assess the injury, the sections were stained with hematoxylin and eosin (HE). To detect collagen accumulation in the tissue, sections were stained with Masson staining, and described using the myocardial collagen volume fraction. ImageJ 1.54p (National Institutes of Health, Bethesda, MD, USA) was used to quantify the collagen area. Sections were observed and photographed under an Olympus BX51 light microscope (Olympus Corporation, Tokyo, Japan).

### 4.4. Cell Culture and Treatment

The embryonic rat heart-derived cell line, H9C2, was purchased from the American Type Culture Collection (ATCC). Cultures were grown at 37 °C in a constant temperature incubator with 5% CO_2_ using complete medium containing high-glucose DMEM, 10% FBS, and 1% penicillin–streptomycin. H9C2 cells were pretreated with exogenously added SESN2 (200 nM, Sigma Aldrich, St. Louis, MO, USA) for 4 h and then stimulated with 400 μM palmitate (PA, Sigma Aldrich, St. Louis, MO, USA, palmitate and BSA solvent are mixed in a 5:1 ratio) for 24 h to mimic high-fat stimulation and induce inflammation. SESN2 protein was dissolved in sterile phosphate-buffered saline (PBS, pH 7.4) containing 0.1% bovine serum albumin (BSA). This concentration and pretreatment duration were adopted according to well-established protocols in H9C2 cells [20,41].

### 4.5. Western Blotting

Heart tissue or cells were added to a lysate mixture containing protein phosphatase inhibitors and RIPA and homogenized under an ice bath. Uniform concentrations were determined with BCA kit proteins, and total proteins were subsequently extracted and separated on SDS-PAGE gels before being transferred to PVDF membranes, and then they were cut into different bands according to the desired molecular weight. Membranes were blocked with 5% skim milk powder for 1 h and then incubated overnight at 4 °C with the following primary antibodies: TNF-α (abcam, ab6671 1:1000), IL-1β (abcam, ab2105, 1:800), IL-6 (abcam, ab233706, 1:1000), IL-10 (CST, 12163S, 1:1000), SESN2 (CST, 8487S, 1:1000), p-AMPK (CST, 2535S, 1:1000), AMPK (CST, 2532S, 1:1000), and PGC-1α (abcam, ab191838, 1:1000). The next day, the membrane was incubated with the appropriate secondary antibody for 1 h at room temperature, followed by luminescence imaging in a gel imaging system using an enhanced chemiluminescence kit.

### 4.6. Reverse Transcription–Polymerase Chain Reaction (RT-qPCR)

Total RNA was isolated using Trizol reagent (Life Technologies, Carlsbad, CA, USA) according to the manufacturer’s protocol. RNA concentration and purity were assessed by measuring the optical density at 260 and 280nm. Purified total RNA was reverse transcribed using a reverse transcription kit (Takara Bio, Kusatsu, Shiga, Japan) and quantitative real-time PCR was performed using SYBR Green premix (Takara, Japa). Quantification of relative gene expression was calculated by the comparative Ct method (2^−ΔΔct^) as described by the manufacturer. The primer sequences were as follows: HMOX1 F: 5′-AGCTTTATGAGGAGTTGCAGGAG-3′ R: 5′-GGTGAAGCACATCCAGAAGG-3′; IL-10 F: 5′-AAGCTCCAAGACCAAGGTGTC-3′ R: 5′-TCCGTTAGCTAAGATCCCTGG-3′; IL-6 F: 5′-CCAGTTGCCTTCTTGGGACT-3′, R: 5′-GGTCTGTTGGGAGTGGTATCC-3′; and B-actin F: 5′-CTGCGTTTTACACCCTTTCTTTG-3′ R: 5′-GCCATGCCAATGTTGTCTCTTAT-3′.

### 4.7. ELISA

Heart tissues or cells were added to phosphate-buffered saline, ground to a homogenate, and centrifuged to collect the supernatant. The total superoxide dismutase (T-SOD) and catalase (CAT) activities and malondialdehyde (MDA) content were measured by commercially available kits purchased from the Nanjing Jiancheng BioEngineering Institute (Nanjing, China). The release of pro-inflammatory cytokines TNF-α and IL-1β in H9C2 cells was measured by ELISA kits (Elabscience Biotechnology Inc Co., Ltd., Wuhan, China). Lactate dehydrogenase was used to assess the damage caused by the high-fat diet on the heart and palmitic acid on H9C2 cells, as measured by the LDH kit (Elabscience Biotechnology Inc. USA, E-EL-M2547).

Detailed steps for the LDH activity measurement are supplemented: LDH levels were measured using a commercial ELISA kit according to the manufacturer’s instructions. Samples and standards (100 μL/well) were incubated at 37 °C for 90 min, followed by washing and incubation with Horseradish Peroxidase (HRP)-conjugate for 60 min at 37 °C. After extensive washing, 3,3′,5,5′-Tetramethylbenzidine (TMB) substrate was added and incubated for 15 min at 37 °C in the dark. The reaction was stopped, and absorbance was read at 450 nm. LDH concentrations were calculated from the standard curve.

### 4.8. Intracellular Reactive Oxygen Species (ROS)

ROS generation was assessed with the fluorescent probe DCFH-DA. H9C2 cells (1 × 10^5^ cells/well) were seeded on coverslips in 6-well plates, pre-treated with SESN2 for 2 h, and then exposed to PA for an additional 24 h. Following the experimental protocol, cells were incubated with DCFH-DA for 30 min. Then, the fluorescence was observed and analyzed using an Olimpus fluorescence microscope.

### 4.9. Statistical Analysis

All experimental data were analyzed and processed by the SPSS22.0 software, and all results were expressed as mean ± standard error (M ± SE). The different groups were compared by one-way analysis of variance followed by Tukey post hoc analysis. Western blotting results were analyzed and processed by the ImageJ software, and the results were plotted by the GraphPad Prism 10.0 software. *p* < 0.05 was considered statistically significant.

## 5. Conclusions

This study demonstrates that treadmill aerobic exercise provides comprehensive protection against high-fat diet-induced obesity myocardial injury through multi-level regulatory mechanisms. Aerobic exercise significantly upregulates the expression of SESN2 and activates the AMPK-PGC-1α signaling pathway, which is potentially involved in alleviating myocardial inflammation, oxidative stress, cardiac fibrosis and cardiac dysfunction in HFD mice. Collectively, our findings highlight treadmill aerobic exercise as a potent and non-pharmacological strategy for ameliorating myocardial injury in HFD-induced obese mice and provide mechanistic evidence supporting its therapeutic potential.

## Figures and Tables

**Table 1:** Echocardiographic indices of mice.

	CON	EX	HFD	FHD + EX
EF (%)	68.1 ± 3.4	73.5 ± 2.8 *	62.0 ± 3.0 ^##^	67.5 ± 2.8 ^^
FS (%)	36.8 ± 2.0	40.5 ± 1.8 *	32.5 ± 2.0 ^#^	36.0 ± 1.5 ^
LVIDd (mm)	3.77 ± 0.18	3.50 ± 0.15	4.00 ± 0.20	3.75 ± 0.16
LVIDs (mm)	2.36 ± 0.20	2.10 ± 0.15	2.70 ± 0.18	2.45 ± 0.16
HR (bpm)	480 ± 15	455 ± 12 *	510 ± 18 ^##^	475 ± 14 ^^

EF: Left ventricular ejection fraction, FS: left ventricular fractional shorting, LVIDd: left ventricular internal dimension diastolic, LVIDs: left ventricular internal dimension systole, HR: heart rate. Values are mean ± SEM. * *p* < 0.05 vs. CON; ^#^ *p* < 0.05 vs. CON; ^##^ *p* < 0.01 vs. CON; ^ *p* < 0.05 vs. HFD; ^^ *p* < 0.01 vs. HFD. *n* = 6.

## Data Availability

The original contributions presented in this study are included in the article Further inquiries can be directed to the corresponding author.

## Abbreviations

The following abbreviations are used in this manuscript:

Sestrin2	SESN2
HFD	High-fat diet
AMPK	AMP-activated protein kinase
PGC-1α	Peroxisome Proliferator-Activated Receptor Gamma Coactivator-1α
ROS	Reactive oxygen species
mTOR	mammalian Target of Rapamycin
HE	Hematoxylin and eosin
TNF-α	Tumor Necrosis Factor-α
IL-1β	Interleukin-1β
IL-6	Interleukin-6
IL-10	Interleukin-10
T-SOD	Total superoxide dismutase
CAT	Catalase
MDA	Malondialdehyde
HRP	Horseradish Peroxidase
TMB	3,3′,5,5′-Tetramethylbenzidine
PBS	Phosphate-buffered saline
BSA	Bovine serum albumin
CVF	Collagen volume fraction
CK-MB	Creatine Kinase-MB
cTnI	Cardiac Troponin I
LDH	Lactate dehydrogenase
HMOX1	Heme Oxygenase1
PA	Palmitate
